# Ancient DNA Analysis Affirms the Canid from Altai as a Primitive Dog

**DOI:** 10.1371/journal.pone.0057754

**Published:** 2013-03-06

**Authors:** Anna S. Druzhkova, Olaf Thalmann, Vladimir A. Trifonov, Jennifer A. Leonard, Nadezhda V. Vorobieva, Nikolai D. Ovodov, Alexander S. Graphodatsky, Robert K. Wayne

**Affiliations:** 1 Department of Genomic Diversity and Evolution, Institute of Molecular and Cellular Biology, Siberian Branch of the Russian Academy of Sciences, Novosibirsk, Russia; 2 Division of Genetics and Physiology, Department of Biology, University of Turku, Turku, Finland; 3 Conservation and Evolutionary Genetics Group, Estación Biológica de Doñana (Consejo Superior de Investigaciones Científicas), Seville, Spain; 4 Institute of Archaeology and Ethnography, Siberian Branch of the Russian Academy of Sciences, Novosibirsk, Russia; 5 Department of Ecology and Evolutionary Biology, University of California, Los Angeles, California, United States of America; University of York, United Kingdom

## Abstract

The origin of domestic dogs remains controversial, with genetic data indicating a separation between modern dogs and wolves in the Late Pleistocene. However, only a few dog-like fossils are found prior to the Last Glacial Maximum, and it is widely accepted that the dog domestication predates the beginning of agriculture about 10,000 years ago. In order to evaluate the genetic relationship of one of the oldest dogs, we have isolated ancient DNA from the recently described putative 33,000-year old Pleistocene dog from Altai and analysed 413 nucleotides of the mitochondrial control region. Our analyses reveal that the unique haplotype of the Altai dog is more closely related to modern dogs and prehistoric New World canids than it is to contemporary wolves. Further genetic analyses of ancient canids may reveal a more exact date and centre of domestication.

## Introduction

The domestication of dogs from the grey wolf is well accepted [1]. However, the timing, location and number of domestication events is still actively debated [2]–[5]. The archaeological record provides unequivocal dog remains beginning about 14,000 calendar years (cy) ago [6]–[7] requiring a domestication that predates agriculture. Putative dog remains ranging in age from 31,000 to 36,000 cy [2] [8]–[9] have been questioned as potentially representing aborted attempts at domestication, or morphologically unique wolves [4]. A full mitochondrial genome analysis of modern dogs suggests an origin in southern China around 16,000 years ago [10], whereas an extensive nuclear genome-wide SNP analysis supports a Middle East and European origin [11], which is more in accordance with archaeological data. Here we isolated, sequenced and analysed 413 nucleotides of the mitochondrial DNA control region from a putative dog specimen dated as approx. 33,000 cy from the Altai Mountains in central Asia. Only a single specimen - namely the Goyet dog (36,000 cy [2]) predates the Altai dog and hence it is thus far the second oldest known specimen assigned morphologically to the domestic dog [8].

## Materials and Methods

### Sample

**Figure 1:**
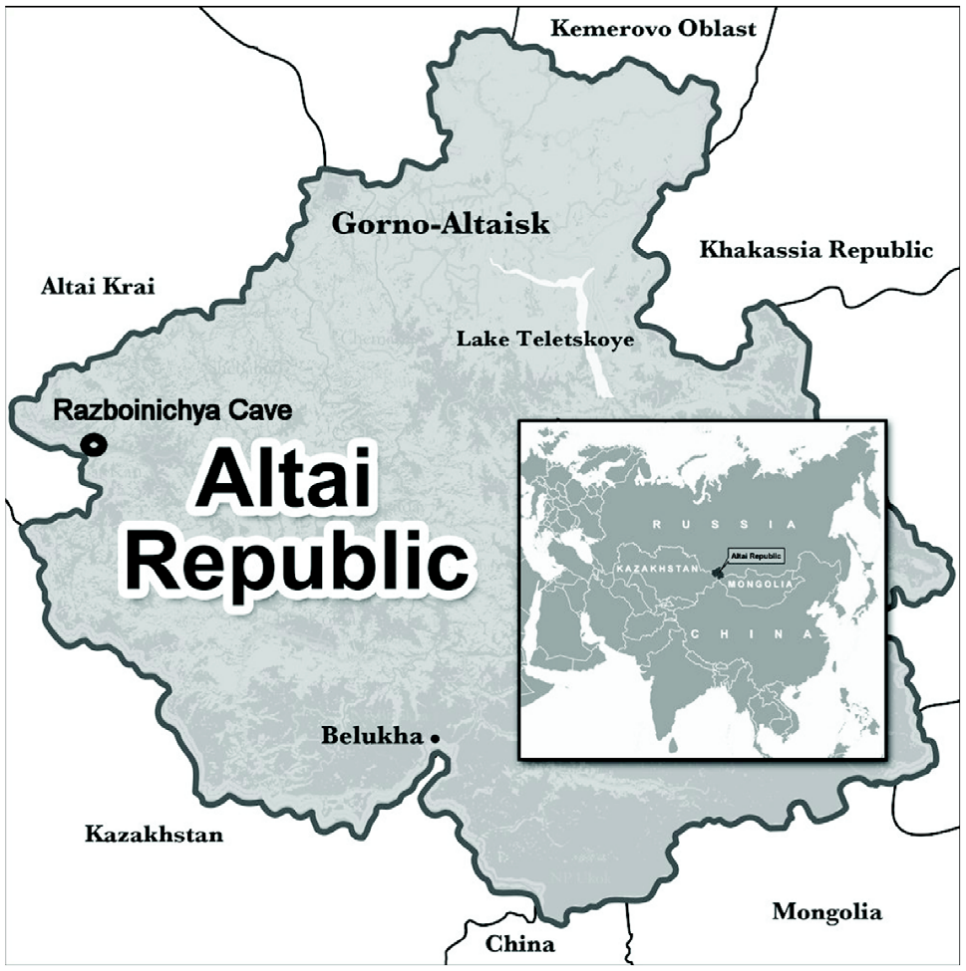
Map depicting the geographic origin of the Altai dog specimen.

The skull of a 33,000 cy old dog-like canid used in this study was excavated from Razboinichya Cave (Altai Mountains of southern Siberia, Figure 1) in 1975 as described previously [8]. The excavation was fully authorized by government authorities and taken place under appropriate grants from the USSR Academy of Sciences. Currently, the sample is part of the collection of the Institute of Archaeology and Ethnography SB RAS. Tooth and mandibular fragments from this sample were provided by Dr. N.D. Ovodov, co-author and member of this Institute, who had institutional authorization to provide this material.

### DNA Extraction, Amplification and Sequencing

We extracted DNA from a right lower lateral incisor and a mandibular bone fragment from the dog-like canid. We conducted two independent extractions of ancient DNA as previously described [12] with some modifications. All extraction steps followed stringent criteria necessary for guaranteeing the authenticity of ancient DNA [13]–[14] such as working in a separate, isolated laboratory and using measures to avoid and detect contamination including negative controls (e.g. extraction mix without any bone material). The surface layer of the bone (0.5–1.0 mm) was removed with a drill and the inner bone material was ground in a mortar into a fine powder. One to 1.5 g of the powder was re-suspended in 15 ml of 0.5 M EDTA pH 8.0, 0.5% N-lauryl sarcosyl (Sigma-Aldrich, Germany) and 0.5 mg/ml proteinase K. The suspension was incubated for 15 min at 55°C. Undissolved pieces were removed by centrifuging at 5,000 g for 5 min. The first fraction was removed and the remaining solid parts were again incubated at 55°C until all bone material was dissolved. The supernatant was concentrated using Amicon Ultra-15 concentrator (Millipore, Germany) with an exclusion size of 5 kDa to a volume of 100–120 µl. The product was purified using the QIAquick PCR Purification Kit (Qiagen, Germany) according to the manufacturer's instructions.

Freshly isolated ancient DNA was amplified using the Whole Genome Amplification (WGA) Kit (Sigma-Aldrich, Germany) as previously described [15].We applied the method to increase the initial amount of aDNA in order to make it available for subsequent experiments. The primers for the amplification of the mitochondrial control region of the ancient dog were designed based on a previously published analyses [16]–[17]. However, we extended the primers’ length based on mitochondrial DNA sequence of *Canis familiaris* (dog *EU789784)* and *Canis lupus* (wolf FJ978035) from GenBank, so we could increase the annealing temperature of the PCR. Primers are listed in Table S1. The location of primers relative to EU789784 GenBank reference sequence is shown in Figure S1. The PCR product length varied from 170 to 389 bp. To prevent amplification of deaminated cytosines common in ancient DNA, we used the Phusion High-Fidelity DNA Polymerase (Thermo Scientific, Finland) following the manufacturer's instructions [18]. For each PCR we used standard concentrations of buffer (manufacturer-provided), dNTPs (0.2 mM), primers (1 µM) and enzyme (0.02 U/µl). One µl of the DNA (WGA product) was added to the 25 µl of the reaction mixture. The PCR protocol included 35 cycles of 3 min at 98°C, 30 sec at 70°C, and 10 sec at 72°C. After two rounds of PCR amplification, PCR residuals were separated by gel electrophoresis and removed by band excision. DNA from the band containing PCR products was isolated using the Gel Extraction Kit (Qiagen, Germany). Sequencing with the same primers used for amplification was carried out in the Inter-institutional center of DNA sequencing at SB RAS.

### Data Analyses

The sequence was first compared to sequences publicly available at the NCBI nr database by means of BLASTN searches [19]. In order to perform Likelihood mapping [20], network [21]–[22], and phylogenetic tree reconstructions, we incorporated the sequence of the Altai specimen into an alignment of 72 dogs (70 known breeds), 30 wolves and four coyotes (Table S2). In addition to contemporary dogs and wolves, we further included sequence information from 35 prehistoric New World canid specimens [23]–[24]. We assessed the substitution model best suited for our dataset by using the model testing option implemented in the program MEGA 5 [25]. We applied either the best model or well supported alternatives whenever the most likely model was not available in the programs used for phylogenetic reconstruction.

Likelihood mapping [20] allows for visualization of a sequence alignment’s phylogenetic informativeness. Utilizing quartet topology weighting, this method provides a powerful tool to evaluate if sequence evolution occurred in a star-shape fashion or resulted in a completely resolved tree. Moreover, by predefining sequence clusters, the method can be used to assess the support of each of the possible topologies in a quartet. Here, we performed Likelihood mapping using TREE-PUZZLE v5.2 [26]. All possible quartets were used when inferring the support of different topologies for various cluster arrangements. However, when assessing the complete dataset without partitioning, only 10,000 quartets were applied. The HKY [27] with five gamma rate categories was used as a substitution model.

The program NETWORK v4.610 was used to construct a phylogenetic network of all haplotypes [21]. An advantage of this phylogenetic reconstruction is a comprehensive visualization of complex haplotype relationships by creating a network instead of a tree and including potential ancestral or intermediate nodes. In order to reduce the complexity of the network, identical haplotypes were combined and their respective frequencies are indicated by the size of the circles in Figure S3. We adjusted the transition/transversion rate by giving five times more weight to transversions and rooted the network with coyotes. We combined the median-joining algorithm with the MP option to further reduce the complexity of the derived network by excluding superfluous links [22].

Lastly, we used the complete mitochondrial genomes of all contemporary dogs and wolves in order to maintain statistically well supported clusters of all dogs and wolves (see Figure S4) and evaluated the relative position of the Altai specimen by means of Neighbour-Joining, Maximum Parsimony and Maximum Likelihood tree reconstructions employing MEGA 5 [25]. In order to evaluate the robustness and the statistical support of the trees, we ran each phylogenetic analysis with 1,000 bootstraps but varied the tree searching algorithms as well as the substitution models. Genetic distances were estimated under the assumption of Kimura 2 Parameter, the best fit model when applying a truncated alignment of 413 nucleotides, and tested for statistical significance using a Mann-Whitney U test implemented in an R-software package.

## Results and Discussion

We obtained mitochondrial DNA control region sequences from both the tooth and mandible of the 33,000 cy old putative dog specimen from Altai and found them to be identical. In order to evaluate the genetic relationship of the Altai specimen to any known dog/wolf specimen, we performed several analyses. First, a BLASTN search of the Altai sequence (413 nucleotides) against the nr database of NCBI revealed similarity at the 99% level (max. score 756), but no perfect match to any extant dog or wolf. Since the sequence was found to be unique, it was deposited in GenBank under accession number JX173682.

Second, we compared our sequence to three previously published 57-nucleotide fragments of wolf mitochondrial DNA from the same cave (Razboinichya cave, 32,500, 48,000 and 50,000 uncalibrated years, [28]) and found the Altai dog to be different at 2, 3 and 3 nucleotide positions, respectively. This indicates that the previously described Pleistocene wolves from the Razboinichya cave are not closely related to the specimen studied here. However, more data of prehistoric wolves from the same region are needed to estimate the population diversity and obtain a more comprehensive picture of genetic relationships of Altai canids.

**Figure 2:**
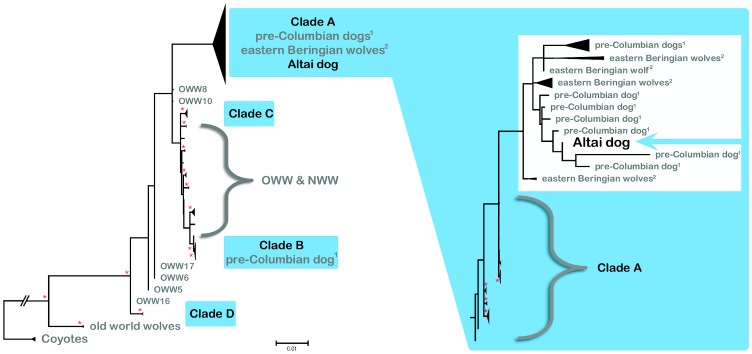
Consensus Neighbour Joining tree (1,000 bootstrap steps) built assuming the Tamura-Nei substitution model, the best fit model for the dataset comprising complete mitochondrial genomes from coyotes (Coyotes), wolves (OWW, NWW – Old and New World wolves, respectively) and dogs combined with partial control region sequences from the Altai specimen (Altai dog) and additional prehistoric canids (pre-Columbian dogs, eastern Beringian wolves). We highlighted all clades containing modern dogs in light blue and enlarged Clade A for better visibility. The position of the Altai specimen is marked with a light blue arrow in the enlargement. Bootstrap values are shown with an asterisk whenever larger than 50.

**Figure 3:**
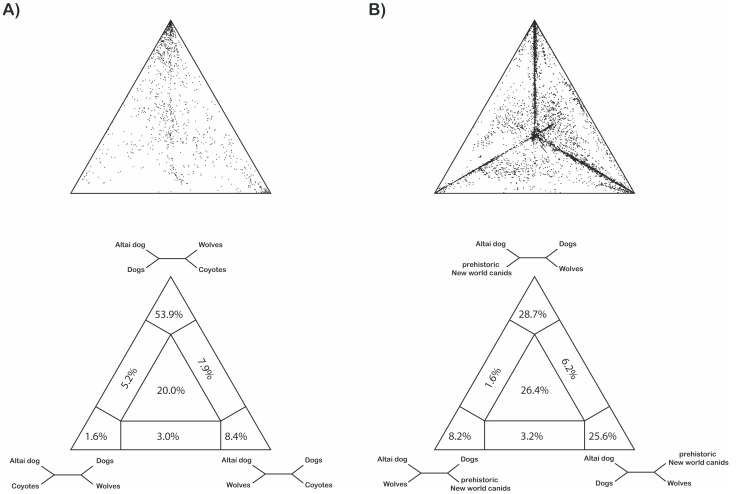
Likelihood mapping analysis off all 142 canid sequences clustered into four groups. Upper panel shows the distribution pattern of all quartets and the lower panel depicts the fraction of each occupied region. Quartets situated in the centre part of the triangle support a star-shaped sequence evolution whereas quartets in the three corners support resolved topologies, respectively. A) Likelihood mapping pattern when clustering the sequences into Altai dog, dogs, wolves and coyotes. B) Likelihood mapping pattern when sequences were clustered as follows: Altai dog, dogs, wolves and prehistoric New World canids taken from [23]–[24].

Unequivocal phylogenetic reconstructions of the evolutionary history of contemporary dogs and wolves have often been hampered by hybridization events within the genus *Canis* [29], leading to unresolved trees or low support values of branching patterns whenever mitochondrial information was utilized (e.g. [1] [30]). When investigating the phylogenetic informativeness of our dataset combining the Altai specimen, 72 extant dogs and 30 wolves, 35 prehistoric New World canids and four coyotes we also found low support for either a clearly resolved branching pattern or star-shaped evolution (Figure S2). The distribution pattern of the 10,000 quartets investigated (Figure S2A) revealed an equal probability of three resolved topologies (23.5%, 23.8% and 23.7% in Figure S2B) and only a marginally lower probability for a star-shaped evolution in which all three topologies are equally likely (21.8%). This result is further supported in haplotype networks which demonstrated star-shaped patterns of divergence (e.g. H_1 (including the Altai specimen), H_48 or H_2 (Figure S3)). Strimmer and Haeseler [20] suggest that analysis of longer sequences may increase the informativeness of the alignment and accordingly, we then compared the Altai sequence to the complete mitochondrial genomes from 72 dogs, 30 wolves and four coyotes as well as overlapping sequences from a total of 35 Pre-Columbian dogs and Pleistocene canids [23]–[24]. The pairwise deletion option applied in the phylogenetic analysis allowed us to retain the haplotype clustering based on complete mitochondrial genomes of contemporary dogs (Figure 2, see also Figure S4, Figure S5), and to place the Altai haplotype with respect to the major dog clades. The bootstrapped Neighbour-Joining tree of ancient and modern sequences shows a well-supported separation of all contemporary dogs from coyotes and two basal wolf haplotypes (Figure 2). The haplotype derived from the Altai specimen clusters among sequences obtained from pre-Columbian dogs and other Late Pleistocene wolf-like canids [23]–[24]. This haplotype cluster is embedded in a clade comprising exclusively contemporary dog sequences (Clade A) and contains the majority of dog haplotypes (45 out of 72) including diverse breeds such as Tibetian Mastiff, Newfoundland, Chinese Crested, Cocker Spaniel or Siberian Husky (Table S2). Notably, the statistical support of the phylogenetic analyses is weak as bootstrap values are low (values below 50 are omitted but see Figure S5). However, although the arrangement of individual haplotypes changed whenever applying different tree building methods (Maximum Likelihood, Maximum Parsimony, Neighbour-Joining), the close relationship and positioning of the Altai haplotype within dog Clade A was consistent. This relationship is further supported by additional analyses such as a four-cluster Likelihood mapping (Figure 3) or the haplotype network (Figure S3). The Likelihood mapping reveals strong support for a topology clustering the Altai specimen with contemporary dogs (Figure 3A: 53.9%) in favour of arrangements grouping the Altai specimen with either coyotes or wolves (Figure 3A: 1.6%, 8.4%, respectively). When exchanging the coyotes with prehistoric New World canids from [23]–[24], topologies uniting the Altai specimen with contemporary dogs or prehistoric canids were almost equally likely. However, these associations were approximately four times more probable than an arrangement of the Altai specimen with modern wolves.

**Figure 4:**
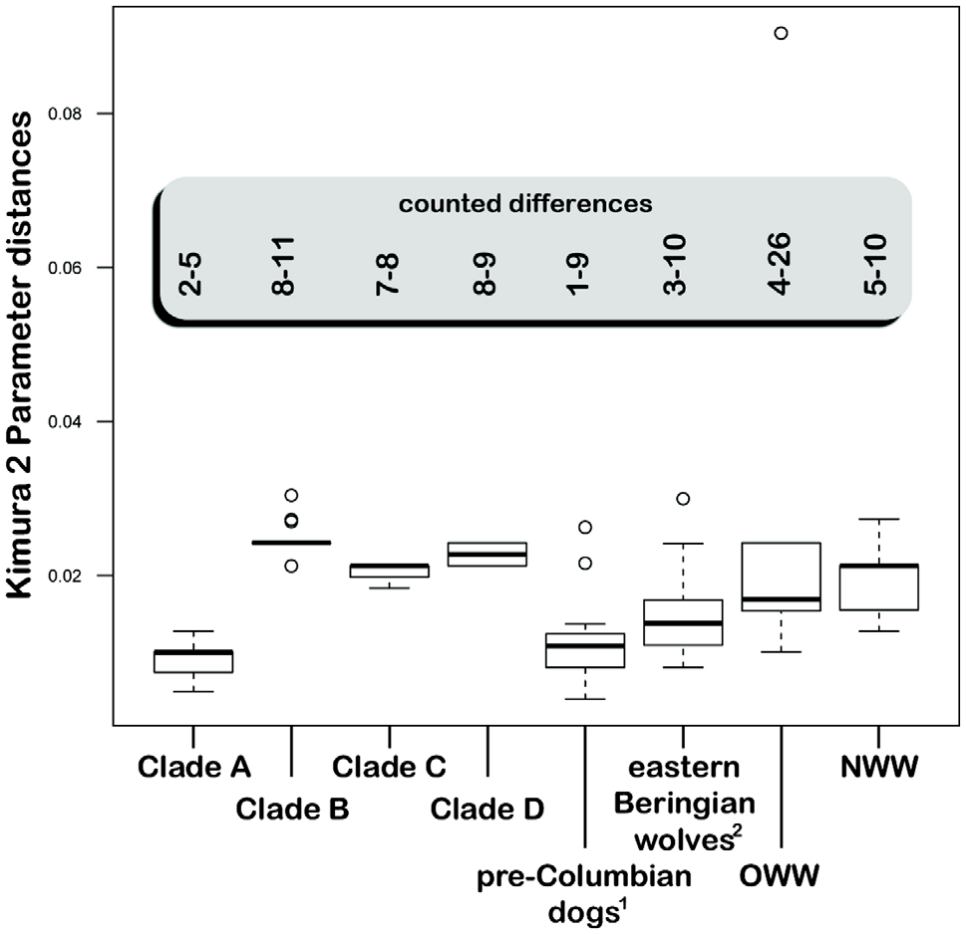
Box plot showing the genetic differences between the Altai specimen and various groupings of contemporary and extinct canids. Genetic distances were estimated under the assumption of Kimura 2 Parameter substitution model, the best fit for the truncated alignment of canids. The highlighted box shows the minimum and maximum counted differences. ^1^ Leonard et al. 2007 [24]; ^2^ Leonard et al. 2002 [23].

A more thorough inspection of the haplotype network also reveals that haplotypes having the closest relationship to the Altai specimen (embedded in H_1 in Figure S3) consist of dog or prehistoric New World canid haplotypes (Figure S3, Table S3). In order to further evaluate these close relationships we analysed the pairwise genetic distance, assuming a Kimura 2 Parameter substitution model (Figure 4) and found that haplotypes clustering in Clade A had the smallest genetic distance (2–5 differences on an alignment of 413 nucleotides) followed by the second smallest distances existing in comparisons to pre-Columbian dogs (1–9 differences), and the largest distance to contemporary wolves (4–26 differences). Moreover, when comparing distances derived from all dog haplotypes to that from all wolves, we found a significantly smaller genetic distance of the Altai specimen to modern dogs (Mann-Whitney U test, p<0.001).

In conclusion, our analyses support the hypothesis that the Altai specimen is more closely related to domestic dogs than to extant wolves, but we stress the point that these analyses were limited to a single, maternally inherited locus and more sequence data would be needed to obtain a statistically well supported phylogeny and unambiguously resolve the genetic relationship of the Altai specimen. However, this preliminary analysis affirms the conclusion that the Altai specimen is likely an ancient dog with a shallow divergence from ancient wolves. These results suggest a more ancient history of the dog outside the Middle East or East Asia, previously suggested as centres of dog origin. Additional discoveries of ancient dog-like remains are essential for further narrowing the time and region of origin for the domestic dog [5].

## Supporting Information

Figure S1 **The scheme of Altai dog sequencing superimposed on the canine mitochondrial DNA sequence from GenBank (EU789784).** Vertical dashed lines indicate the boundaries of 413 bp sequence used in this work. Small arrows indicate positions of all primers from Table S1. Bars 1–6 indicate independent PCR reactions with different primer combinations: (1) – D1F/D1R (365 bp); (2) – D2F/D09R (389 bp); (3) – D1F/D2R (343 bp); (4) – D10F/D09R (195 bp); (5) – D3F/D3R (212 bp); (6) – D5F/D09R (170 bp).
(TIF)

Figure S2 **Likelihood mapping analysis of all 142 canids without further partitioning of the data.** Upper panel shows the distribution pattern of all quartets and the lower panel depicts the fraction of each occupied region.
(PDF)

Figure S3 **Haplotype Network summarizing the phylogenetic relationships of unique haplotypes.** Identical haplotypes are collapsed and the sizes of the circles indicate the frequencies. Different haplotypes are labelled with H_XX (yellow circles) and hypothesized median vectors with mvXX (red circles). The length of the links between nodes is proportional to mutational differences. For better visibility, the link to the root (coyotes) and two aberrant wolf-haplotypes are truncated and the haplotype group containing the Altai dog is highlighted in green.
(PDF)

Figure S4 **Neighbour Joining tree generated with complete mitochondrial genomes of 72 dogs and 30 wolves using 1,000 bootstrap steps.** The shaded areas indicate the four well supported dog clusters and the arrows point at the support values for each clade.
(PDF)

Figure S5 **Neighbour Joining tree representing a fully annotated version of the tree shown in** **Figure 2** **generated with 413 bp of the hypervariable region of the mitochondrial genome.** Red values indicate the bootstrap support after 1,000 steps. The identifiers are explained in Table S2 with the exception of sequences labelled “JAL”. The latter nomenclature is adopted from Leonard et al. 2007 [24] and Leonard et al. 2002 [23] and the arrows point at the support values for each clade.
(PDF)

Table S1 **List of primers used in this study.**
(PDF)

Table S2 **Nomenclature, accession number and breed/geographical origin of the individual dog/wolf haplotypes.**
(PDF)

Table S3 **Haplotype assignments for the network analysis.**
(PDF)
