# Supplementary figures and images for: Ancient DNA Analysis Affirms the Canid from Altai as a Primitive Dog
Source: PLoS One. 2013 Mar 6;8(3):e57754. doi: 10.1371/journal.pone.0057754 (PMC3590291; doi:10.1371/journal.pone.0057754)

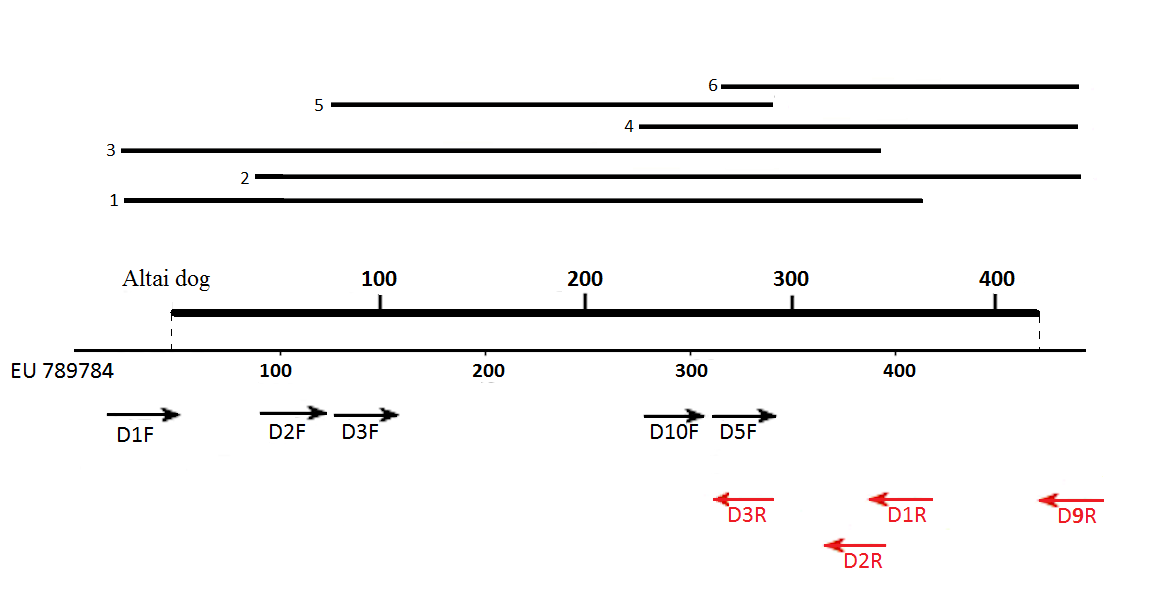

Supplement: Figure S1 — The scheme of Altai dog sequencing superimposed on the canine mitochondrial DNA sequence from GenBank (EU789784). Vertical dashed lines indicate the boundaries of 413 bp sequence used in this work. Small arrows indicate positions of all primers from Table S1. Bars 1–6 indicate independent PCR reactions with different primer combinations: (1) – D1F/D1R (365 bp); (2) – D2F/D09R (389 bp); (3) – D1F/D2R (343 bp); (4) – D10F/D09R (195 bp); (5) – D3F/D3R (212 bp); (6) – D5F/D09R (170 bp). (TIF) [file pone.0057754.s001.tif]

A)

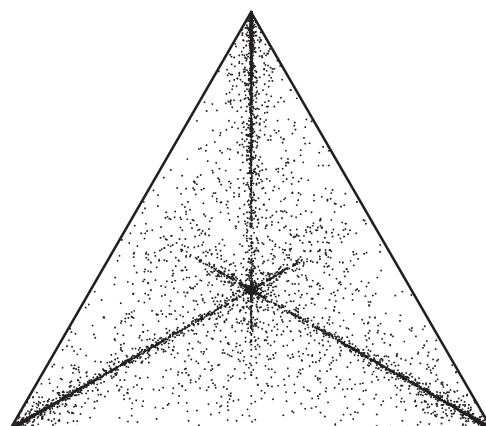

B)

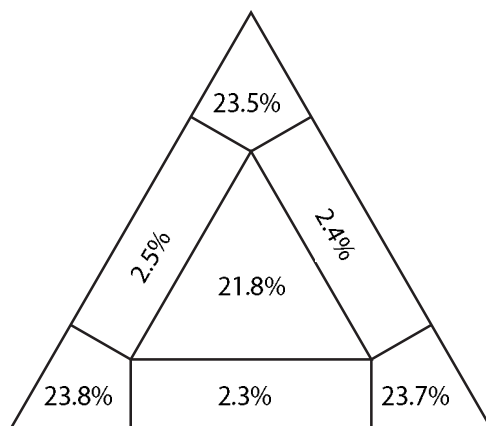

Supplement: Figure S2 — Likelihood mapping analysis of all 142 canids without further partitioning of the data. Upper panel shows the distribution pattern of all quartets and the lower panel depicts the fraction of each occupied region. (PDF) [file pone.0057754.s002.pdf]

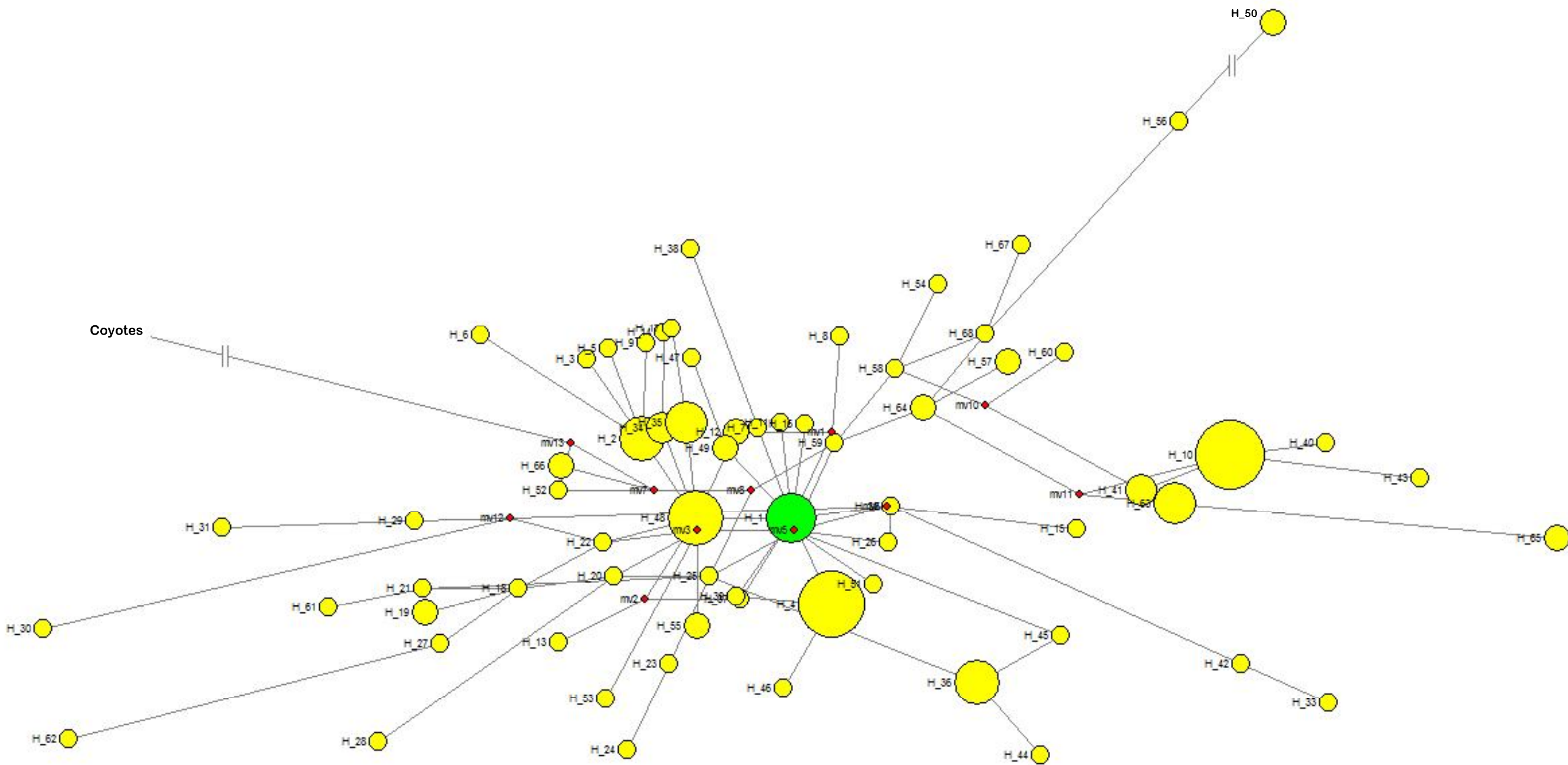

Supplement: Figure S3 — Haplotype Network summarizing the phylogenetic relationships of unique haplotypes. Identical haplotypes are collapsed and the sizes of the circles indicate the frequencies. Different haplotypes are labelled with H_XX (yellow circles) and hypothesized median vectors with mvXX (red circles). The length of the links between nodes is proportional to mutational differences. For better visibility, the link to the root (coyotes) and two aberrant wolf-haplotypes are truncated and the haplotype group containing the Altai dog is highlighted in green. (PDF) [file pone.0057754.s003.pdf]

D - Dog haplotypes  
OWW - Old world wolves  
NWW - New world wolves

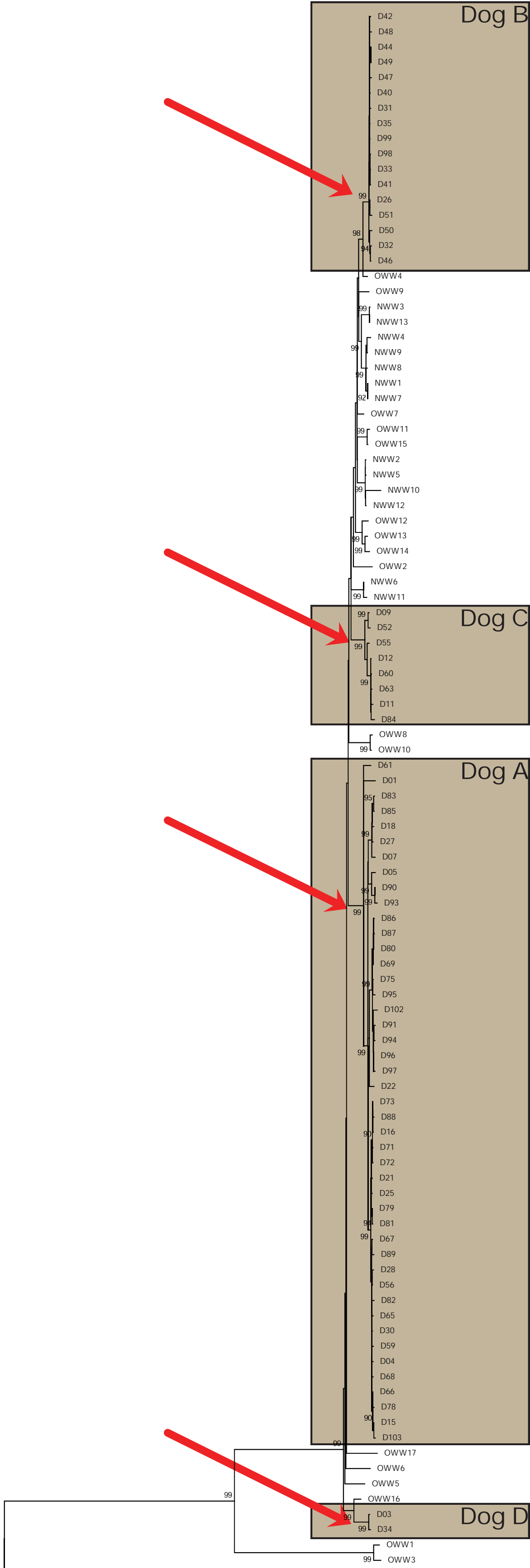

Supplement: Figure S4 — Neighbour Joining tree generated with complete mitochondrial genomes of 72 dogs and 30 wolves using 1,000 bootstrap steps. The shaded areas indicate the four well supported dog clusters and the arrows point at the support values for each clade. (PDF) [file pone.0057754.s004.pdf]

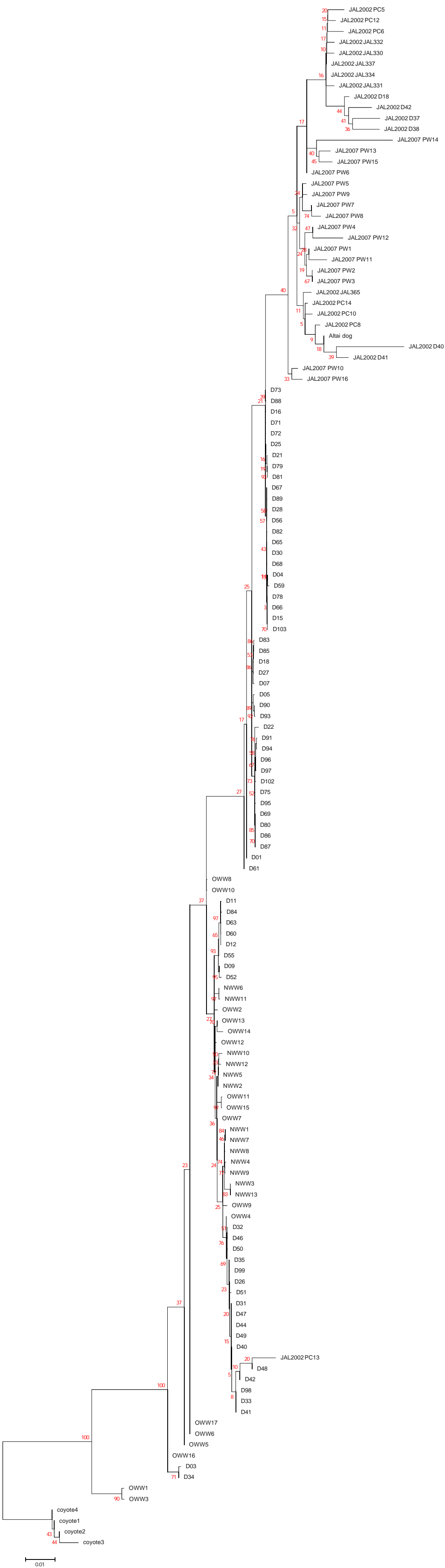

Supplement: Figure S5 — Neighbour Joining tree representing a fully annotated version of the tree shown in Figure 2 generated with 413 bp of the hypervariable region of the mitochondrial genome. Red values indicate the bootstrap support after 1,000 steps. The identifiers are explained in Table S2 with the exception of sequences labelled “JAL”. The latter nomenclature is adopted from Leonard et al. 2007 [24] and Leonard et al. 2002 [23] and the arrows point at the support values for each clade. (PDF) [file pone.0057754.s005.pdf]
