# Supplementary material for: Ancient DNA Analysis Affirms the Canid from Altai as a Primitive Dog
Source: PLoS One. 2013 Mar 6;8(3):e57754. doi: 10.1371/journal.pone.0057754 (PMC3590291; doi:10.1371/journal.pone.0057754)
Supplement: Table S1 — List of primers used in this study. (PDF) [file pone.0057754.s006.pdf]

**Supplementary Table 1**
List of primers used in this study

| The primers used for amplification of canine mitochondrial DNA control region | | |
|---|---|---|
| Name | Sequence | The annealing temperature 100mM NaCl |
| D1F | GCTCTTGCTCCACCATCAGCACC | 68,8 |
| D2F | TTCCCTGACACCCCTACATTCATATATTG | 66.9 |
| D3F | CCCCTACTGTGCTATGTCAGTATCTCCAG | 67,1 |
| D5F | GTCCAATAAGGGCTTAATCACCATGCC | 69,1 |
| D10F | TCCAATAAGGGCTTAATCACCATGCC | 68.6 |
| D1R | GTAACCCCCACGTTAGTATGGGCCC | 69.8 |
| D2R | GCCCGGAGCGAGAAGAGGGAC | 70.0 |
| D3R | GGCATGGTGATTAAGCCCTTATTGGAC | 69.1 |
| DO9R | TCCATCGAGATGTCCCATTTGCGA | 67.7 |
