# Supplementary material for: Ancient DNA Analysis Affirms the Canid from Altai as a Primitive Dog
Source: PLoS One. 2013 Mar 6;8(3):e57754. doi: 10.1371/journal.pone.0057754 (PMC3590291; doi:10.1371/journal.pone.0057754)
Supplement: Table S2 — Nomenclature, accession number and breed/geographical origin of the individual dog/wolf haplotypes. (PDF) [file pone.0057754.s007.pdf]

Supplementary Table 2

Nomenclature, Accession number and breed/geographical origin of the individual dog/wolf haplotypes.

Dog clade A		
ID	Accession number	Breed
1	DQ480499	Siberian Husky
4	DQ480495	Cocker Spaniel
5	DQ480497	West Highland White Terrier
7	DQ480491	Irish Setter
15	AY656755	Sapsaree
16	AY656753	Irish Setter
18	AY656741	Irish Setter
21	AY656754	Chinese Crested
22	AY656737	Basenji
25	AY656744	English Springer Spaniel
27	AY656749	Saint Bernard
28	AY656742	Old English Sheepdog
30	AY656747	Welsh Springer Spaniel
56	EU408305	Viszla
59	EU408282	Keeshond
61	EU408300	Tibetan Mastiff
65	EU408272	Dachshund
66	EU408270	Dachshund
67	EU408261	Chihuahua
68	EU408246	American Cocker Spaniel
69	EU408304	unknown
71	EU408295	Rottweiler
72	EU408286	Miniature Dachshund
73	EU408249	Australian Shepherd
75	EU408274	English Mastiff
78	EU408248	Australian Shepherd
79	EU408264	Cairn Terrier
80	EU408294	Pug
81	EU408287	Newfoundland
82	EU408302	Toy Poodle
83	EU408290	Neapolitan Mastiff
85	EU408289	Neapolitan Mastiff
86	EU408263	Cavalier King Charles Spaniel
87	EU408250	Bichon Frise
88	EU408266	Cocker Spaniel
89	EU408273	English Shepherd
90	EU408275	French Bull Dog
91	EU408265	Corgi
93	EU408276	Great Dane
94	EU408245	Akita
95	EU408257	Brittany Spaniel
96	FJ817358	Golden Retriever
97	FJ817362	Golden Retriever
102	FJ817359	Golden Retriever
103	U96639	Sapsaree

Dog clade B		
ID	Accession number	Breed
26	AY656743	Saint Bernard
31	AY656752	Standard Schnauzer
32	AY656745	English Springer Spaniel
33	AY656740	Kerry Blue Terrier
35	EU408269	Doberman Pinscher
40	EU408252	Bolognese
41	EU408292	Poodle
42	EU408260	Cardigan Corgi
44	EU408254	Basset Hound
46	EU408307	Walker Hound
47	EU408303	unknown
48	EU408247	Australian Terrier
49	EU408255	Basset Hound
50	EU408268	Cocker Spaniel
51	EU408258	Cockapoo
98	FJ817364	Golden Retriever
99	FJ817363	Golden Retriever

Dog clade C		
ID	Accession number	Breed
9	DQ480489	German Shepherd
11	DQ480501	Swedish Elkhound
12	DQ480493	Black Russian Terrier
52	EU408293	Pit Bull Terrier
55	EU408251	Blue Heeler
60	EU408267	Cocker Spaniel
63	EU408279	Havanese
84	EU408291	Pomeranian

Dog clade D		
ID	Accession number	Breed
3	DQ480492	Jamthund
34	EU408288	NorwegianElkhound

OWW		
ID	Accession number	Origin
OWW1	NC011218	China
OWW2	GQ374438	Mongolia
OWW3	EU442884	China
OWW4	AM711902	Sweden
OWW5	DQ480507	Saudi Arabia
OWW6	DQ480506	Saudi Arabia
OWW7	DQ480505	Spain
OWW8	Thalmann unpublished	Finland
OWW9	Thalmann unpublished	Russia
OWW10	Thalmann unpublished	Russia
OWW11	Thalmann unpublished	Sweden
OWW12	Thalmann unpublished	China
OWW13	Thalmann unpublished	Israel
OWW14	Thalmann unpublished	India
OWW15	Thalmann unpublished	Russia
OWW16	Thalmann unpublished	Poland
OWW17	AB499824	Japan

NWW		
ID	Accession number	Origin
NWW1	Thalmann unpublished	Canada
NWW2	Thalmann unpublished	Canada
NWW3	Thalmann unpublished	Alaska
NWW4	DQ480508	Canada
NWW5	Thalmann unpublished	Canada
NWW6	Thalmann unpublished	Mexico
NWW7	Thalmann unpublished	Canada
NWW8	Thalmann unpublished	Canada
NWW9	Thalmann unpublished	Canada
NWW10	Thalmann unpublished	USA
NWW11	Thalmann unpublished	Mexico
NWW12	Thalmann unpublished	Alaska
NWW13	Thalmann unpublished	Alaska
