# Supplementary material for: Ancient DNA Analysis Affirms the Canid from Altai as a Primitive Dog
Source: PLoS One. 2013 Mar 6;8(3):e57754. doi: 10.1371/journal.pone.0057754 (PMC3590291; doi:10.1371/journal.pone.0057754)
Supplement: Table S3 — Haplotype assignments for the network analysis. (PDF) [file pone.0057754.s008.pdf]

Supplementary Table 3

Haplotype assignments for Network analysis

Unique haplotype in Fig. S2	# sequences	Names													
Hap_1*	7	Altai	D16	D25	D71	D72	D73	D88							
Hap_2	6	JAL2002_JAL330	JAL2002_JAL331	JAL2002_JAL334	JAL2002_JAL337	D91	D94								
Hap_3	1	JAL2002_JAL332													
Hap_4	13	JAL2002_JAL365	D04	D15	D30	D56	D65	D66	D67	D68	D78	D82	D89	D103	
Hap_5	1	JAL2002_PC5													
Hap_6	1	JAL2002_PC6													
Hap_7	1	JAL2002_PC8													
Hap_8	1	JAL2002_PC10													
Hap_9	1	JAL2002_PC12													
Hap_10	14	JAL2002_PC13	D26	D33	D35	D40	D41	D42	D44	D47	D48	D49	D98	D99	OWW4
Hap_11	1	JAL2002_PC14													
Hap_12	2	JAL2002_D18	D01												
Hap_13	1	JAL2002_D37													
Hap_14	1	JAL2002_D38													
Hap_15	1	JAL2002_D40													
Hap_16	1	JAL2002_D41													
Hap_17	1	JAL2002_D42													
Hap_18	1	JAL2007_PW1													
Hap_19	2	JAL2007_PW2	JAL2007_PW3												
Hap_20	1	JAL2007_PW4													
Hap_21	1	JAL2007_PW5													
Hap_22	1	JAL2007_PW6													
Hap_23	1	JAL2007_PW7													
Hap_24	1	JAL2007_PW8													
Hap_25	1	JAL2007_PW9													
Hap_26	1	JAL2007_PW10													
Hap_27	1	JAL2007_PW11													
Hap_28	1	JAL2007_PW12													
Hap_29	1	JAL2007_PW13													
Hap_30	1	JAL2007_PW14													
Hap_31	1	JAL2007_PW15													
Hap_32	1	JAL2007_PW16													
Hap_33	1	D03													
Hap_34	3	D05	D90	D93											
Hap_35	5	D07	D18	D27	D83	D85									
Hap_36	6	D09	D11	D12	D60	D63	D84								
Hap_37	1	D21													
Hap_38	1	D22													
Hap_39	1	D28													
Hap_40	1	D31													
Hap_41	3	D32	D46	D50											
Hap_42	1	D34													
Hap_43	1	D51													
Hap_44	1	D52													
Hap_45	1	D55													
Hap_46	1	D59													
Hap_47	1	D61													
Hap_48	9	D69	D75	D80	D86	D87	D95	D96	D97	D102					
Hap_49	2	D79	D81												
Hap_50	2	OWW1	OWW3												
Hap_51	1	OWW2													
Hap_52	1	OWW5													
Hap_53	1	OWW6													
Hap_54	1	OWW7													
Hap_55	2	OWW8	OWW10												
Hap_56	1	OWW9													
Hap_57	2	OWW11	OWW15												
Hap_58	1	OWW12													
Hap_59	1	OWW13													
Hap_60	1	OWW14													
Hap_61	1	OWW16													
Hap_62	1	OWW17													
Hap_63	5	NWW1	NWW4	NWW7	NWW8	NWW9									
Hap_64	2	NWW2	NWW5												
Hap_65	2	NWW3	NWW13												
Hap_66	2	NWW6	NWW11												
Hap_67	1	NWW10													
Hap_68	1	NWW12													
Hap_69	1	coyote1													
Hap_70	2	coyote2	coyote4												
Hap_71	1	coyote3													

* Haplotype group containing the Altai specimen

Grey shades indicate Haplotype groups closest to the Altai specimen
